# Preregistration in practice: A comparison of preregistered and non-preregistered studies in psychology

**DOI:** 10.3758/s13428-023-02277-0

**Published:** 2023-11-10

**Authors:** Olmo R. van den Akker, Marcel A. L. M. van Assen, Marjan Bakker, Mahmoud Elsherif, Tsz Keung Wong, Jelte M. Wicherts

**Affiliations:** 1 Department of Methodology and Statistics, Tilburg University, Warandelaan 2, 5037 AB Tilburg, The Netherlands; 2 Department of Sociology, Utrecht University, Utrecht, The Netherlands; 3 Department of Neuroscience, Psychology, and Behaviour, University of Leicester, Leicester, UK

**Keywords:** Effect size, HARKing, P-hacking, Preregistration, Positive results, Research impact

## Abstract

Preregistration has gained traction as one of the most promising solutions to improve the replicability of scientific effects. In this project, we compared 193 psychology studies that earned a Preregistration Challenge prize or preregistration badge to 193 related studies that were not preregistered. In contrast to our theoretical expectations and prior research, we did not find that preregistered studies had a lower proportion of positive results (Hypothesis 1), smaller effect sizes (Hypothesis 2), or fewer statistical errors (Hypothesis 3) than non-preregistered studies. Supporting our Hypotheses 4 and 5, we found that preregistered studies more often contained power analyses and typically had larger sample sizes than non-preregistered studies. Finally, concerns about the publishability and impact of preregistered studies seem unwarranted, as preregistered studies did not take longer to publish and scored better on several impact measures. Overall, our data indicate that preregistration has beneficial effects in the realm of statistical power and impact, but we did not find robust evidence that preregistration prevents *p*-hacking and HARKing (Hypothesizing After the Results are Known).

## Introduction

Researchers often hypothesize the presence of a causal effect or association between two or more variables. When a study shows evidence for such an effect or association, the result is typically branded as “positive.” Conversely, when a study does not show such evidence, the result is typically branded as “negative.” Although finding a positive result is not necessarily the result of better scholarship, positive results are more likely to be published (Dickersin, 1990; Ferguson & Brannick, 2012; Franco et al., 2014) and are more often cited (Duyx et al., 2017) than negative results. Moreover, peer reviewers more often recommend articles with positive results for publication than those with negative results because they think positive results contribute more to science (Mahoney, 1977), and researchers write up or submit positive results for publication more often than negative results because they think positive results have more publication potential (Franco et al., 2014). Further evidence of a bias against negative results comes from studies that find that the vast majority of results in the scientific literature are positive (Dickersin et al., 1987; Sterling, 1959), particularly in psychology (Fanelli, 2010), despite the common use of underpowered designs (Bakker et al., 2012). It appears that academics perceive studies with positive results as more valuable than studies with negative results,^1^ possibly because the dominance of significance testing in many fields (e.g., Hubbard, 2015) leads researchers to equate positive results with significance.

The premium on positive results may also shape the behavior of academics in other ways. While carrying out a study, researchers may, consciously or unconsciously, steer their study towards a positive result. Two main examples of this are HARKing (Hypothesizing After the Results are Known (Bosco et al., 2016; John et al., 2012; Kerr, 1998; Motyl et al., 2017) and *p*-hacking (John et al., 2012; Motyl et al., 2017). When researchers HARK, they misattribute a research result to a certain theory *after* distilling the results from the data, which is problematic because one can almost always find something of interest in a given dataset with many variables. When researchers *p*-hack, they make research decisions *contingent on* their data, often with the aim of achieving a *p*-value below .05. These so-called questionable research practices (QRPs) artificially create positive results, as the data do not always warrant the conclusion that an association between variables exists (Murphy & Aguinis, 2019; Simmons et al., 2011).

To prevent researchers from engaging in HARKing and *p*-hacking, it has been suggested that researchers post their hypotheses, study design, and analysis plan online before collecting or looking at any data (Nosek et al., 2018; Wagenmakers et al., 2012). This practice is called preregistration and would help to avoid HARKing because publicizing a study’s hypotheses before data are collected makes it impossible for researchers to pretend that they theorized the study results beforehand. Similarly, preregistration would help avoid *p*-hacking because researchers have to specify most of their research decisions before data collection, restricting their freedom to make these decisions contingent on the data. Because preregistration theoretically prevents HARKing and *p*-hacking, preregistered publications should contain a lower proportion of positive results than non-preregistered publications (Hypothesis 1). This study aimed to test this hypothesis for publications in psychology.

A positive result may not be the only desirable outcome. The same may be said for a large effect size, since large effect sizes indicate associations of a higher magnitude and thus more convincing evidence (Kelley & Preacher, 2012). Researchers may therefore want to *p*-hack their way to a larger effect size in a similar way as they would to a positive result (Fanelli et al., 2017; Ioannidis, 2008). Based on that conjecture, we also predicted that effect sizes are on average larger in non-preregistered than in preregistered studies (Hypothesis 2). This predicted effect could be driven by the premium on large effect sizes but could also be a by-product of the premium on positive results. It could also be that non-preregistered studies have larger effect sizes because they have smaller sample sizes, as positive results require larger effect sizes to be statistically significant in smaller studies (see also Hypothesis 5 below).

Three recent studies directly compared preregistered publications with non-preregistered publications in psychology. First, Schäfer and Schwarz (2019) found a lower proportion of positive results (0.64 vs. 0.79) and lower median effect sizes (0.16 vs. 0.36) in preregistered publications (including registered reports, a type of preregistration where studies are peer-reviewed *before* data collection; see Chambers & Tzavella, 2022) than in non-preregistered publications. They did not compare the proportion of positive results in published registered reports and “regular” preregistered publications but found similar mean effect sizes in published registered reports and “regular” preregistered publications (0.18 vs. 0.22). Second, Scheel et al. (2021) found a lower proportion of positive results in published registered reports than in non-preregistered publications (0.44 vs. 0.96). However, the authors did not compare the magnitudes of effect sizes. Finally, Toth et al. (2021) found that preregistered studies (including registered reports) included a lower proportion of positive results (0.48) than non-preregistered studies (0.66). Additionally, they investigated some other differences between preregistered studies and non-preregistered studies. In line with Bakker et al. (2020), they found that preregistered studies more often reported a sample size rationale than non-preregistered studies (proportions of 0.72 vs. 0.29), but such rationales were not associated with larger sample sizes. A final result from Toth et al. shows that preregistered studies were more likely to discuss excluded data (0.78 vs. 0.51) and were more likely to have an a priori stopping rule (0.43 vs. 0.02).

Our project differs from these previous studies in four ways. First, we only compared “regularly” preregistered studies to non-preregistered studies, and thus excluded registered reports. Excluding registered reports allows for a purer assessment of the effect of preregistration, as registered reports also differ from non-preregistered studies in that these reports are adjusted based on peer review.

Second, while two earlier studies did not specifically match preregistered and non-preregistered studies, we linked each preregistered study in our sample to an equivalent non-preregistered study. More specifically, Scheel et al. (2021) used a random sample of 152 psychology publications by searching for the string “test the hypothesis” in the Web of Science Essential Science Indicators (ESI) database. Schäfer and Schwarz (2019) used a stratified random sample of 900 publications, 10 randomly selected from each of 90 journals that were themselves randomly selected from Web of Science subject categories within psychology (10 per category, but none for mathematical psychology). Only Toth et al. (2021) matched preregistered and non-preregistered studies, by using a combination of (1) non-preregistered studies in papers with an included preregistered study, and (2) non-preregistered studies in papers from the same journal issue (or the same year) as the included preregistered study. In our study, we looked at Web of Science’s list of related papers (based on the number of overlapping references) for every preregistered publication and selected the first non-preregistered publication in this list with empirical data that was published in the same year as the preregistered publication. This ensured that the preregistered and non-preregistered publications (broadly) matched on topic and publication period.

Third, rather than coding a limited set of hypotheses as in the three earlier studies, we aimed to code *all* hypotheses in a study. Scheel et al. (2021) selected only the result of the first hypothesis mentioned in a paper that was explicitly tested, Schäfer and Schwarz (2019) selected the first result related to the key research question, and Toth et al. (2021) selected the results of all hypotheses but only if they were formally stated. In our study, we took a more inclusive approach by assessing the first statistical result for *all hypotheses* in a paper, including those that were not formally stated (i.e., hypotheses that were not listed but could be found in the running text of a preregistration). We had already identified hypotheses and the corresponding statistical results from preregistered studies a priori as part of another project (Van den Akker et al., 2023b, see also https://osf.io/z4awv).

Finally, we extend earlier studies by looking at other variables on top of effect size and the proportion of positive results and examining whether they differ between preregistered and non-preregistered publications.

In a survey about QRPs, John et al. (2012) asked a sample of psychology researchers whether they ever “rounded off” a *p*-value (e.g., reported a *p*-value of .054 as less than .05). They found that a little over 20% admitted to having done so at least once, and studies screening the psychological literature indeed found that half of all papers reporting significance tests contained at least one inconsistent *p*-value (Nuijten et al., 2016). The 20% rate of admission is relatively low compared to the other QRPs in the John et al. (2012) survey. Interestingly, however, the authors also found that a respondent’s admission to a relatively rare QRP, such as incorrectly rounding off *p*-values, predicted that the respondent also engaged in other QRPs, such as failing to report all of the study’s dependent measures or deciding to collect more data after checking whether the results were significant. Incorrectly rounding off *p*-values is a QRP that cannot be prevented by preregistration, but based on the finding by John et al. (2012), it may be a proxy of QRPs that *can* be prevented by preregistration like outcome switching or optional stopping (Wicherts, 2017). We therefore expected that incorrectly reported *p*-values would be less prevalent in preregistered publications than in non-preregistered publications (Hypothesis 3).

The main benefit of preregistration is that it prevents HARKing and *p*-hacking, but preregistration also comes with other benefits (Lakens, 2019; Sarafoglou et al., 2022). First and foremost, preregistering a study requires careful deliberation about the study’s hypotheses, research design, and statistical analyses. This deliberation might be spurred on by researchers’ use of preregistration templates that provide guidance on what to include in a preregistration and why (e.g., Bowman et al., 2016; Haven & Van Grootel, 2019; Van den Akker et al., 2021). For example, many preregistration templates stress the importance of performing a proper power analysis to determine the study’s sample size. We therefore expected that the sample sizes of preregistered studies would be based on power analyses more often than the sample sizes of non-preregistered studies (Hypothesis 4).

Moreover, because studies without a power analysis often rely on sample size rationales that lead to relatively low statistical power (Bakker et al., 2016), we expected that the sample sizes in preregistered studies would be larger than the sample sizes in non-preregistered studies (Hypothesis 5). Indeed, Schäfer and Schwarz (2019) found that preregistered publications involved larger sample sizes than non-preregistered publications, and Maddock and Rossi (2001) showed that studies requiring a power analysis as part of a federal funding scheme had higher power to detect medium and small effects than other studies. On the other hand, Bakker et al. (2020) found that studies based on preregistration templates recommending power analyses did not have larger sample sizes than studies based on preregistration templates not recommending power analyses.

Some researchers have voiced worries that it is more difficult for preregistered studies than non-preregistered studies to get published. For example, researchers have expressed concerns that the restrictive nature of preregistration leads to boring or messy papers without room for unexpected discoveries (Goldin-Meadow, 2016; Kornell, 2013; and see Giner-Sorolla, 2012), making it harder to get them published. However, one could also argue that preregistered studies are more likely to be published because their perceived trustworthiness may make studies with negative results more appealing. Because we do not have information about non-published preregistrations, it is difficult to investigate whether preregistered studies are harder to publish than non-preregistered studies. However, many journals do provide information about the duration of reviews. For preregistered studies, peer review should involve a comparison of the preregistration to the final manuscript, which may cause the review process to take longer. On the other hand, preregistered papers may be of higher quality or may be more clearly reported, which could result in fewer review rounds and a shorter review process. For this reason, we did not have a clear hypothesis about the association between preregistration and review duration, but we did examine this exploratively.

We also assessed the scientific impact of preregistered publications versus non-preregistered publications. To that end, we looked at three well-known metrics: a publication’s number of citations, a publication’s Altmetric Attention Score, and the impact factor of the publishing journal. The number of citations and the journal impact factor have traditionally been key markers of scientific impact (Mingers & Leydesdorff, 2015). The Altmetric Attention Score is relatively new and takes into account less traditional measures of impact such as references in news outlets, on blogs, and on social media like Facebook and Twitter (see https://www.altmetric.com/about-our-data/the-donut-and-score for more information). Scientometric studies largely found positive relationships between traditional citation counts and both separate altmetrics (micro-blogging: *r* = .003, blogs: *r* = .12, bookmarks from online reference managers: *r* = .23 for CiteULike, and *r* = .51 for Mendeley; Bornmann, 2015) and the Altmetric Attention Score (*r* = .23; Huang et al., 2018). We had no a priori hypothesis about the association between preregistration and these three indicators of scientific impact.

### Hypotheses

1. Preregistered studies have a lower proportion of positive results than similar non-preregistered studies.
2. Preregistered studies contain smaller effect sizes than similar non-preregistered studies.
3. Preregistered studies have a lower proportion of gross statistical inconsistencies than similar non-preregistered studies.
4. Preregistered studies more often contain a power analysis than similar non-preregistered studies.
5. Preregistered studies contain larger sample sizes than similar non-preregistered studies.

## Method

### Sample of preregistered studies

Our sample of preregistered studies was derived from a large-scale project that investigated selective hypothesis reporting (Van den Akker et al., 2023b) that included published papers that earned a Preregistration Challenge prize and published papers that earned a preregistration badge prior to 2020. The Preregistration Challenge was a campaign organized from 2017 to 2018 by the Center for Open Science (COS) where researchers could earn $1000 when they published a preregistered study. Preregistration badges were also initiated by the COS; journals could decide to hand out preregistration badges to papers that included at least one preregistered study. After excluding registered report studies, studies using secondary data, and studies using nonhuman subjects, the earlier project included a sample of 459 preregistered studies from 259 papers.

For the current project, we only included studies for which a preregistered statistical result was retrievable in the running text, and we included only the first study of a paper to prevent dependency in the data. This led to a final sample size of 208 studies, which deviates from our preregistered sample of 210 for the following reasons. After preregistering, we noticed that one study had no retrievable result (Banks et al., 2018), while another study involved changes to the preregistration after review, technically qualifying it as a registered report (Goldberg & Carmichael, 2017). While extracting the required information for the remaining 208 papers, we had to exclude an additional 12 papers because they were published in journals that were not listed in the Web of Science Core Collection (which we used to find a control paper, see below), namely *Comprehensive Results in Social Psychology*, *Psi Chi Journal of Psychological Research*, *BMC Psychology*, and *Wellcome Open Research*. We also had to exclude a paper from *Psychological Science* because we could only find a corrigendum on Web of Science, rather than the actual paper. The list of the 193 remaining studies in our sample is available at https://osf.io/xzcnb.

**Fig. 1:**
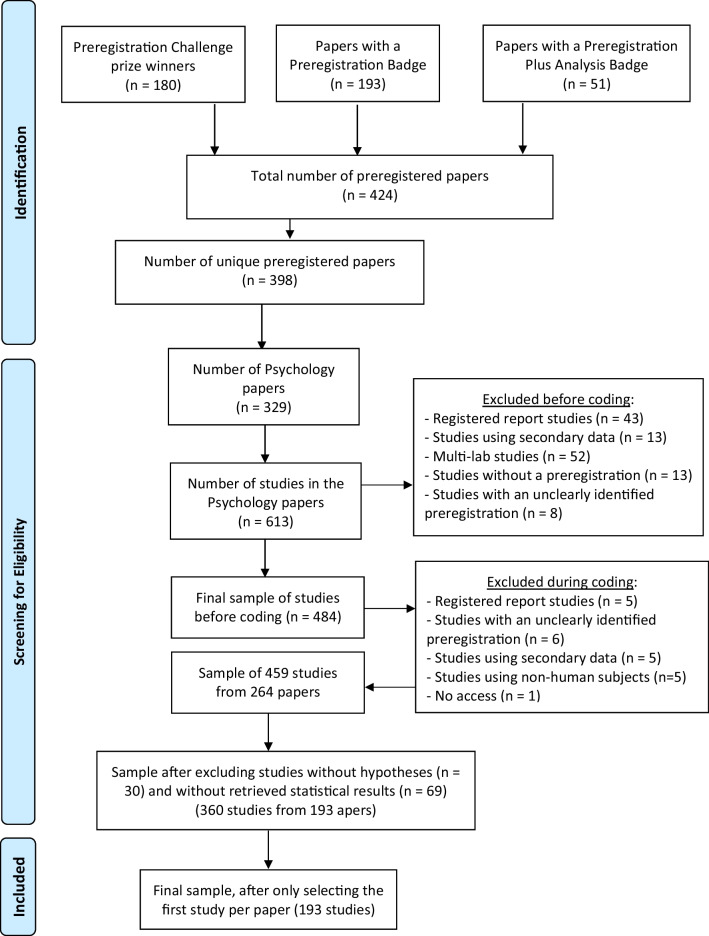
PRISMA flow diagram outlining the full sample selection procedure

The data collection procedure is detailed in Van den Akker et al. (2023b), and an overview of the procedure can be found in the PRISMA flow diagram (Moher et al., 2009) in Fig. 1.

### Sample of non-preregistered studies

To create a control group for comparison with the preregistered studies in our sample, we linked each preregistered publication in our sample to a non-preregistered publication. We did so by checking Web of Science’s list of related papers for every preregistered publication and selecting the first non-preregistered publication from that list that used primary quantitative data and was published in the same year as the related preregistered publication. To check whether publications were preregistered, we searched the publication for the keyword “regist.” If that keyword could not be found, we assumed that the publication was not preregistered. We chose Web of Science because it covers established peer-reviewed journals and the searches using its Core Collection database are reproducible (in contrast to the searches in Google Scholar, Gusenbauer & Haddaway, 2020) and because comparable studies also used Web of Science (Scheel et al., 2021; Schäfer & Schwarz, 2019), facilitating any comparisons we might want to make.

Our control group was deliberately chosen to mimic our sample of preregistered publications as closely as possible. We preferred this over a random sample of psychology papers because it could be that preregistration is more common in one subfield of psychology than in another. If that is the case, we would compare a skewed sample of preregistered publications to a representative sample of non-preregistered publications. Comparing our sample of preregistered publications to a control group of similar publications is therefore more pertinent. The list of the 193 control studies is available at https://osf.io/xzcnb.

### Assessing whether a hypothesis was supported

To assess the proportion of positive results in preregistered studies, we built on the earlier project on selective hypothesis reporting (Van den Akker et al., 2023b). In that project, hypotheses were identified in both preregistrations and their corresponding papers to see whether selective reporting took place. Hypotheses were identified by using the keywords “replicat,” “hypothes,” "investigat," “test,” “predict,” “examin,” and “expect,” and included whether the authors predicted a relationship between two or more variables using any of these keywords (disregarding manipulation checks and checks of statistical assumptions). We then tried to match a statistical result in the published paper to each of the preregistered hypotheses. If a match was found, we inspected the statistical output and concluded that there was a positive result when *p* < .05, or when Bayes factors (BFs) were either smaller than 1/3 or larger than 3. If multiple results matched the preregistered hypothesis, we chose the first statistical result mentioned in that paper. If the authors specifically stated that they used a significance level smaller than .05 or a Bayes factor criterion smaller than 1/3 or larger than 3, we used the authors’ inference criteria. The end result was a fraction: the number of hypotheses with a matched positive result divided by the total number of matched results. If no *p*-value or Bayes factor could be retrieved, we coded this as missing data (“NA”). The protocol for the assessment of the support for preregistered hypotheses can be found at https://osf.io/fdmx4 (for preregistrations) and https://osf.io/uyrds (for publications).

We extracted the proportion of positive results for our sample of non-preregistered publications by inspecting the results sections of these publications and flagging all statistical results that were not part of a manipulation check, a check of statistical assumptions, or an exploratory test. Using all the flagged results in a paper, we calculated the proportion of positive results by assessing *p*-values and Bayes factors as we did for preregistered publications.

### Effect sizes

We use the Fisher-transformed Pearson’s *r* as our common effect size measure because it was found to be the most frequently reported effect size in Schäfer and Schwarz (2019) and because its interpretation is relatively straightforward. If *r* was not specified for a certain result, we calculated it based on the *t*-value or *F*-value and the accompanying degrees of freedom. If the *F*-statistic was based on multiple contrasts or variables (*df*_1_ > 1), we followed the Open Science Collaboration (2012) and computed the “correlation coefficient per degree of freedom” (*r*/*df*_1_). Table 1 provides the formulas we used for these calculations. If a statistical result was not based on a *t*- or *F*-statistic (but on a *z*- or χ^2^-statistic for example) or a statistical result did not include sufficient information to calculate *r*, we did not include the result.

**Table 1:** Formulas used to compute the correlation coefficients per degree of freedom

Statistic	Transformation
*t*	$r=\sqrt{\frac{t^2\ast \frac{1}{df}}{t^2\ast \frac{1}{df}+1}}$
*F*	$r=\sqrt{\frac{F\ast \frac{df_1}{df_2}}{F\ast \frac{df_1}{df_2}+1}}\sqrt{\frac{1}{df_1}}$

*df* = *N* − 1, *df*_1_ = *n*_1_ − 1, *df*_2_ = *n*_2_ − 1

### Reporting errors

We used the *statcheck* web app (Rife et al., 2016) in June 2022 to count the number of “grossly” incorrectly reported *p*-values (i.e., *p*-values that did not match their accompanying test statistic and degrees of freedom *and* for which the inconsistency changed the statistical conclusion; Nuijten et al., 2016) and used the proportion of gross errors per study as our dependent variable. Because we used *statcheck,* we only included results in the analyses that could be extracted using that program (i.e., *t*, *F*, *r*, χ^2^, and *z*-statistics). Exploratively, we also looked at “regularly” incorrectly reported *p*-values (i.e., *p*-values that did not match their accompanying test statistic and degrees of freedom but for which the inconsistency did not change the statistical conclusion).

### Power analysis and sample size

We determined sample size and the presence of a power analysis as part of a project assessing the effectiveness of preregistration (Van den Akker et al., 2023a; see https://osf.io/x7qgh for the coding protocol). Of concern here is the effective sample size (i.e., the sample size that is used to draw conclusions about the hypothesis selected using the hypothesis selection protocol, see https://osf.io/z4aw). We used the same procedure to determine the presence of a power analysis and the sample size for preregistered and non-preregistered publications.

### Review duration

To compare the duration of reviews of preregistered publications and non-preregistered publications, we checked the article history of these publications to extract the submission date and the date of acceptance. The difference between the two in number of days was used as our measure of review duration. One potential issue with this method is that journals may not always accurately register submission dates and acceptance dates. However, we expected any inaccuracies to occur equally frequently for preregistered and non-preregistered publications. We used the same procedure to determine the submission and acceptance dates for preregistered and non-preregistered publications.

### Scientific impact

We coded the number of citations, the journal impact score, and the Altmetric Attention Score all in the same week (May 16–20, 2022). We determined the number of citations by searching for a manuscript on the Web of Science Core Collection database, the 2019 journal impact factor by using Web of Science’s Journal Citation Reports, and the Altmetric Attention Score by using the Altmetric.com bookmarklet. We used the same procedure to determine these metrics for preregistered and non-preregistered publications.

### Hypothesis tests

We tested our preregistered hypotheses (see https://osf.io/mpd3u) with five bivariate regressions in which the independent variable was whether a study was preregistered. The dependent variables in these regressions were the proportion of positive results in the study (Hypothesis 1), Fisher-transformed effect size (Hypothesis 2), the proportion of statistical inconsistencies in the study (Hypothesis 3), the presence of a power analysis in the study (Hypothesis 4), and the log of the sample size of the study (Hypothesis 5). Hypotheses 1, 3, and 4 were tested using logistic regressions. Hypothesis 2 was tested using a multilevel linear regression with two levels: statistic (level 1), and study (level 2). Hypothesis 5 was tested using a linear regression.

Power analyses for the five hypotheses are reported in our preregistration but were based on the planned 210 rather than the actual 193 included studies. Rerunning the power analyses using the same anticipated effect sizes as in our preregistration but using the actual sample size of 193 resulted in a statistical power of 1.00 for Hypothesis 1, 0.97 for Hypothesis 2, 0.83 for Hypothesis 3, and 0.63 for Hypothesis 4. The updated power calculations are available at https://osf.io/m47f6.

The R code for all hypothesis tests can be found at https://osf.io/sujfa. All data used for the analyses can be found at https://osf.io/pqnvr.

## Results

We found no support for Hypothesis 1 that the proportion of positive results was lower in preregistered studies (0.69, *SD* = 0.38) than in non-preregistered studies (0.68, *SD* = 0.25), β = 0.01, 99% CI [−0.56, 0.59], *z*(366) = 0.05, *p* = .96. For this analysis we deviated from our preregistration and excluded all null hypotheses from the sample of preregistered studies. We felt that this was warranted because we realized, in hindsight, that the calculation of the proportion of positive results for non-preregistered studies assumed that all hypotheses were directional. Excluding preregistered null-hypotheses therefore makes the above comparison between preregistered and non-preregistered studies fairer. When we did include the null-hypotheses, as we preregistered, the statistical result was as follows: β = 0.01, 99% CI [−0.57, 0.58], *z*(366) = 0.03, *p* = .98.

We also did not find support for Hypothesis 2. While effect sizes were numerically smaller on average for preregistered (0.29, *SD* = 0.24, median = 0.28) than non-preregistered studies (0.36, *SD* = 0.25, median = 0.30), this difference was not statistically significant, β = −0.04, 99% CI [−0.12, 0.04], *t*(1794.2) = −1.36, *p* = .175.

Hypothesis 3 was also not supported by our data. Preregistered publications (0.001, *SD* = 0.01) did not have a lower proportion of gross statistical inconsistencies than non-preregistered publications (0.005, *SD* = 0.03), β = −1.33, 95% CI [−7.46, 4.80], *z*(216) = −0.42, *p* = .671. When we looked at all statistical inconsistencies (including ones where the statistical conclusion did not change), we also did not find a lower proportion of inconsistencies in preregistered publications (0.03, *SD* = 0.15) than in non-preregistered publications (0.09, *SD* = 0.19), β = −1.19, 95% CI [−2.51, 0.13], *z*(213) = −1.76, *p* = .08.

In line with Hypothesis 4, we found that sample sizes in preregistered studies (0.55) were more often based on a power analysis than sample sizes in non-preregistered studies (0.23), β = 1.38, 99% CI [0.81, 1.95], *z*(383) = 6.17, *p* < .0001. Accordingly, we also found support for Hypothesis 5: the sample sizes of preregistered studies (mean = 959.0, median = 216) were larger than the sample sizes of non-preregistered studies (mean = 536.6, median = 116), β = 0.45, 99% CI [0.14, 0.76], *t*(384) = 3.72, *p* = .0002.

### Preregistered exploratory analyses

We employed four bivariate regressions to explore whether preregistration influenced review duration (Exploration 1), the log of the number of citations (Exploration 2), the log of journal impact factor (Exploration 3), and the log of Altmetric Attention Score (Exploration 4).

We did not find evidence that the review time of preregistered studies (257.9 days, *SD* = 176.6) was different from the review time of non-preregistered studies (269.4 days, *SD* = 213.1), β = −11.54, 95% CI [−54.2, 31.2], *t*(318) = −0.53, *p* = .597.

Interestingly, for measures of scientific impact, the results did highlight an effect of preregistration. Preregistered publications received more citations (18.3, *SD* = 24.6) than non-preregistered publications (15.1, *SD* = 18.4), β = 0.20, 95% CI [0.01, 0.40], *t*(384) = 2.09, *p* = .038, using α = .05. Preregistered publications (103.9, *SD* = 204.0) also received a higher Altmetric Attention Score than non-preregistered publications (28.3, *SD* = 63.0) and were published in journals with a higher impact factor (4.1, *SD* = 1.4 vs. 3.0, *SD* = 1.6), Altmetric score, β = 1.27, 95% CI [0.26, 0.44], *t*(373) = 6.98, *p* < .0001; and impact factor, β = 0.35, 95% CI [0.92, 1.63], *t*(375) = 7.53, *p* < .0001.

## Conclusion and discussion

In this project, we compared studies that earned a Preregistration Challenge prize or preregistration badge with similar studies that were not preregistered. Unexpectedly, we did not find that preregistered studies had a lower proportion of positive results than non-preregistered studies (Hypothesis 1) or that they had smaller effect sizes (Hypothesis 2). Moreover, preregistered studies did not include fewer statistical inconsistencies than non-preregistered studies, as we expected (Hypothesis 3). We did find support for Hypothesis 4 and Hypothesis 5: preregistered studies more often contained a power analysis and had larger sample sizes than non-preregistered studies. Our preregistered exploratory analyses found that there was no difference in review times between the study types, and that preregistered studies had a greater impact in terms of citations, Altmetric Attention Score, and journal impact factor than non-preregistered studies.

The higher statistical power and larger sample sizes in preregistered than non-preregistered studies are important, considering earlier findings that sample sizes across psychology are often insufficient to find meaningful effects (Bakker et al., 2016; Szucs & Ioannidis, 2017). In line with our finding regarding statistical power, Maddock and Rossi (2001) found that federally funded studies (that typically included an a priori power analysis) had higher average power than studies that did not receive such funding (and typically did not include an a priori power analysis). Prior research on the link between a study’s preregistration and sample size is mixed: Schäfer and Schwarz (2019) found that preregistered studies had larger sample sizes than non-preregistered studies for between-subject designs, but smaller sample sizes for within-subject designs.

Not finding an association between preregistration and positive results or effect size contrasts with earlier research (Schäfer & Schwarz, 2019; Scheel et al., 2021; Toth et al., 2021). For the proportion of positive results in preregistered studies, we would expect that our estimate would be higher than earlier estimates that were based on samples including registered report studies. This expectation stems from the idea that regular preregistrations and registered reports both prevent *p*-hacking and HARKing due to increased transparency, but registered reports additionally prevent publication bias because editors accept or reject the paper before the results of the study are known. Insofar as the samples are comparable, our estimate of 0.68 falls, as expected, above prior estimates by Scheel et al. (2021; 0.44, registered reports only), Schäfer and Schwarz (2019; 0.64, registered reports and regularly preregistered studies), and Toth et al. (2021; 0.48, registered reports and regularly preregistered studies). As a robustness check, it would be useful to compare our estimate with the estimates based only on the regularly preregistered studies in Schäfer and Schwarz, and Toth et al., thereby filtering out the influence of registered reports. However, neither study disclosed the particular studies they coded, so this proved impossible.

Surprisingly, the proportion of positive results we found in non-preregistered studies (0.69) was lower than estimates from previous work (Fanelli, 2010: 0.92; Schäfer & Schwarz, 2019: 0.79; Scheel et al., 2021: 0.96; Sterling et al., 1995: 0.96), with one exception (Toth et al., 2021: 0.61). The heterogeneity in the five estimates can be at least partly explained by the different methods that were used to retrieve the statistical results from the non-preregistered studies. Fanelli and Scheel et al. assessed whether authors concluded finding positive (full or partial) or negative (null or negative) support for the first hypothesis in the paper. One explanation for their very high estimates is that they also counted partially positive results as positive, thereby possibly including results with spin (e.g., results that the authors claimed as positive while the *p*-value was marginally significant, see Olsson-Collentine et al., 2019). Schäfer and Schwartz first identified the key research question of a study based on the title and abstract, and then extracted the first reported effect that unambiguously referred to that key research question. As Fanelli, Scheel et al., and Schäfer and Schwarz all focused on the first or key hypothesis in the paper, their high estimates may be explained by a focus on the study’s pivotal hypothesis. In contrast, Toth et al. counted all hypotheses that were formally stated in the introduction section, and for which an explicit statistical conclusion could be found elsewhere in the paper. Similarly, in our study, we extracted all statistical results in the results section of non-preregistered studies except for checks of manipulations and statistical assumptions. We contend that the inclusion of other than pivotal statistical results lowered our and Toth et al.’s estimates of the proportion of positive results.

Our results-oriented approach was chosen because experiences from other projects (Van den Akker et al., 2023a, b) led us to expect that it would be too difficult to find the first or most important hypothesis in non-preregistered studies. While this approach seems inclusive and encompassing, it could be that we included statistical results that were not meant as hypothesis tests. However, we would argue that readers of papers tend to see all results in a results section as hypothesis tests unless they are clearly labeled as a check or as exploratory. Nevertheless, there is currently no well-validated method to assess the proportion of positive results of hypothesis tests in non-preregistered studies. It would help if researchers highlighted in their papers what results in their results section were intended as hypothesis tests, and whether these tests were preregistered.

Another explanation for the similar proportion of positive results in preregistered and non-preregistered studies is that sample sizes were larger for the former (see the results for Hypothesis 5). Assuming true effect sizes are equal for preregistered and non-preregistered studies (which would be in line with the results for Hypothesis 2), we would expect higher statistical power and, thus, a higher proportion of positive results for the preregistered studies. As a crude test of this explanation, we ran the analysis of Hypothesis 1 again but with sample size as a control variable. Controlling for sample size, we again did not find a difference in the proportion of positive results in preregistered and non-preregistered studies, β_1_ *=* 0.0026, *t*(364) = 0.078, *p =* .938, demonstrating that the role of this alternative explanation is probably minor.

Finally, it is important to note that preregistered and non-preregistered publications can differ in yet other aspects. For example, it is likely that researchers self-select to carry out a preregistration, and researchers who preregister may be more junior, more conscientious, or more concerned with abiding by responsible research practices like preregistration. Because of these differences, causal claims about the effect of preregistration on the proportion of positive results or effect size are difficult to make. Future studies may aim to identify the characteristics of preregistering and non-preregistering researchers so that these variables could be included as control variables in studies like ours.

Taking all results together, we conclude that preregistered studies are of higher quality than non-preregistered studies in the sense that they more often contain power analyses than non-preregistered studies and typically have higher sample sizes. Moreover, concerns about the publishability of preregistered versus non-preregistered studies seem unwarranted, as preregistered studies do not take longer to publish and have greater impact. Our study does not provide convincing evidence that preregistration prevents *p*-hacking and HARKing of results reported in the main text of a study, as both the proportion of positive results and effect sizes are similar between preregistered and non-preregistered studies. Future research could shed more light on this. One could, for example, include preregistration as a moderator in meta-analyses on theoretically similar effects. If non-preregistered studies typically involve larger observed effects, this could be an indication of biases (publication bias and/or QRPs). Such empirical work, combined with the results from the current study, would improve our understanding of preregistration and would allow us to make more evidence-based claims about its practical value.

## Footnotes

1 Awareness of this issue has prompted the creation of several journals open to (e.g., *PLOS One*, *F1000*, and *PeerJ*), or even dedicated to publishing negative results (e.g., the *Journal of Articles in Support of the Null Hypothesis*, and the *Journal of Pharmaceutical Negative Results*).
